# The Information and Consent Process in Patients undergoing Elective ENT surgery: A cross-sectional survey

**DOI:** 10.1186/1472-6815-8-5

**Published:** 2008-09-17

**Authors:** Christos Georgalas, Kulandaivelu Ganesh, Eva Papesch

**Affiliations:** 1 Department of Otolaryngology, Whipps Cross University Hospital, London, UK

## Abstract

**Background:**

To assess the importance of different information pathways for patients undergoing elective ENT surgery (General Practitioner, Specialist consultation, pre assessment clinic and consent process as well as printed information material and non medical sources) and to correlate their relative importance with patient and doctor factors

**Methods – Patients:**

Cross – sectional questionnaire survey

226 consecutive patients undergoing elective non-oncological otolaryngology procedures at a District General Hospital between May and August 2004

**Results:**

Overall patients were moderately satisfied with the information they received prior to surgery (score 63/100). Although they were generally satisfied with the quality of information they received at their outpatient consultation and at the preadmission clinic, they were less satisfied with the quality of information provided by their GPs and by the quality of self – obtained information. Most importantly, linear regression modeling showed that the overall level of information could be predicted by three factors: The quality of written information received at the hospital, the quality of self-obtained information and the information provided by the specialist at the time of listing for surgery. While patient's education level was correlated with the information process, the age and gender of the patient as well as the grade of the doctor at the outpatients were not associated with his overall levels of satisfaction.

**Conclusion:**

Although the impact of the initial outpatient consultation for patients undergoing elective surgery can not be over emphasized, written information provided at the hospital as well as patient – initiated, parallel information pathways are at least as important: It is our duty to recognize them and use them for the patient's advantage.

## Background

The acquisition of medical information before undergoing an operation is a complex process: It is increasingly recognised that the patient plays an active role rather than just passively accepts doctors' advice [1,2] and that pre-operative information and preparedness with surgery may correlate with a successful surgical outcome [3] Increasingly, non medical sources of information such as the internet, are being utilized by patients [4]. In this era of increased patient expectations the medical profession is trying to optimise information delivery, by using patient decision software [5], printed information sheets (in up to 25% of ENT departments) [6] and increasingly by shifting the role of provision of patient information to more senior members of the team[7,8]. The patients' information needs and information-seeking behavior is correlated with their age, sex, education and socioeconomic level as well as the nature of illness and surgery performed [9]. On the other hand, there are factors relating to the GP, the operating surgeon and the doctor obtaining the consent and the overall system in place. However, no synthesis has been made until now of all these factors and how they interact to provide a patient with enough information for his surgery. We set up a survey to evaluate these factors and their interaction and to assess from a patients perspective, the role and quality of each information source.

## Methods

### Aim of study

To assess the importance of different information pathways for patients undergoing elective ENT surgery (GP visit, outpatient consultation, pre assessment clinic and consent process as well as printed information material and non medical sources) and to correlate their relative importance with patient and doctor factors.

### Type of study

The study was undertaken in the public health sector. This system is based on a free hospital service, open to patients referred from their General Practitioner (GP). The GP acts as an assessor and information giver, and discuss different options and outcomes with the patient.

This study was a cross-sectional survey, undertaken in a large hospital in London. Patients were asked to grade the quality of the information they received from their GP, the ENT doctor at their outpatient appointment, and the ENT doctor at the preadmission clinic. Although it is understood that the task of informing the patient rests mainly with the ENT specialists, the GP is also expected (within the limits of his expertise) to provide information about the surgery to the patient. An anonymous patient questionnaire (Additional file 1) was completed after their operation but before their discharge, using visual analogue scales (VAS). Patients were also asked to comment on the quality of information contained in the consent form, information sheets supplied and self-obtained information (internet, friend etc). Finally, they were asked to quantify the overall completeness of their information prior to surgery. (See Appendix)

### Sample

A consecutive series of 250 patients undergoing elective ENT surgery, between May 2004 and August 2004 were studied. Nursing staff handed over the questionnaire, with clear instructions on how to complete it, together with the discharge summary and medication. After completion, the questionnaires were returned to a designated folder in the nurse's station. Patients undergoing oncological surgery or whose knowledge of English prevented them from understanding the questionnaire were excluded from the study.

### Ethical issues

Verbal informed consent to participate in the study, was obtained by all the participants in the study. The ethics committee opinion was sought, and they felt that since this was a patient survey, there was no need for a formal ethics committee review.

### Analysis

Descriptive statistical analysis was undertaken of diagnoses, operation performed, age, sex, education level and levels of quality of information. Perceived quality and adequacy of information from various sources was correlated with demographic and medical factors. In order to define the factors predicting overall information quality, we performed linear regression of overall completeness of information using as independent factors different information sources as well as patient and doctor factors.

## Results

A total of 226 patients (90%) returned a completed questionnaire, 48% of which were female. The mean age was 37.8 years (SD 6.8, range 16 to 60). Their median education level was A levels (National exam in the UK, sat at the age of 17–18 years of age) (40%), while 19% had university education and 3% had a postgraduate qualification. The case load was typical of a district general hospital: 48 patients underwent tonsillectomy, 54 had septoplasty, 36 had panendoscopies including microlaryngoscopy, 32 had endoscopic sinus surgery, 17 had major ear surgery, 7 underwent rhinoplasty, 9 had major neck surgery (thyroidectomy, parotidectomy), as well as 3 closures of septal perforation, 3 grommets and 17 other minor surgery. Almost all the operations that the patients underwent (with the exception of the 9 patients undergoing major neck surgery) are classified as of minor/moderate severity and resulted in the patients being discharged the same day or the following day from the hospital.

Informed written consent for the surgery was obtained by the Senior House Officer (SHO) in all cases. The patient was listed for surgery by the Registrar or the Staff Grade Doctor in 62% of cases, by the SHO in 14% (in all cases under the direct supervision of the Consultant) and by the Consultant in 24% of cases.

### Perceived quality of various information sources

**Figure 1:**
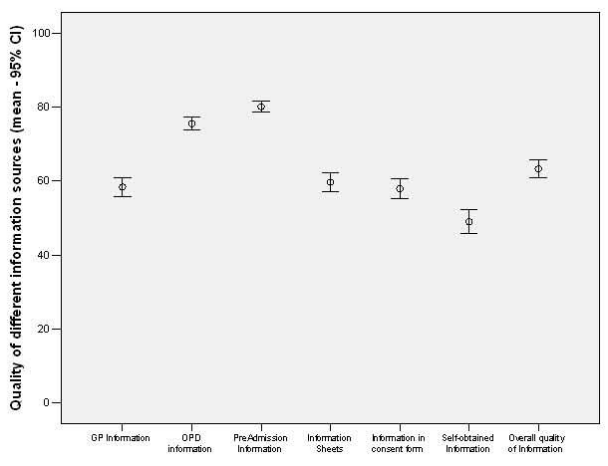
**Patient – rated quality of information from different sources **(Visual analogue scale).

The self-rated quality of information gathered from different sources is shown in Table 1. Overall the patients felt that the information they received prior to their surgery was adequate to good (graded 6.3 in a ten-point-scale). They felt that the quality of information provided at the preadmission clinic and outpatient consultation was highest (8/10 and 7.5/10 respectively), while self obtained/non medical information was considered of relatively low quality (4.9/10) (Figure 1)

**Table 1:** Patient-rated quality of Different Information Sources

	N	Perceived Quality (scale 0 = poor, 100 = excellent)	Standard Deviation
Information supplied by GP	218	58,39	19,17
Specialist Information	223	75,56	12,67
Preadmission clinic	223	80,12	11,42
Information Sheets	215	59,69	18,90
Information contained in consent form	220	57,94	19,83
Self-Obtained Information (internet, friends)	186	49,01	22,73
Overall Information Received	214	63,30	17,754

### Overall satisfaction with information received – univariate analysis

We tried to assess which (extrinsic or intrinsic) factors were closely associated with the outcome of a well informed patient:

### Patient factors

**Figure 2:**
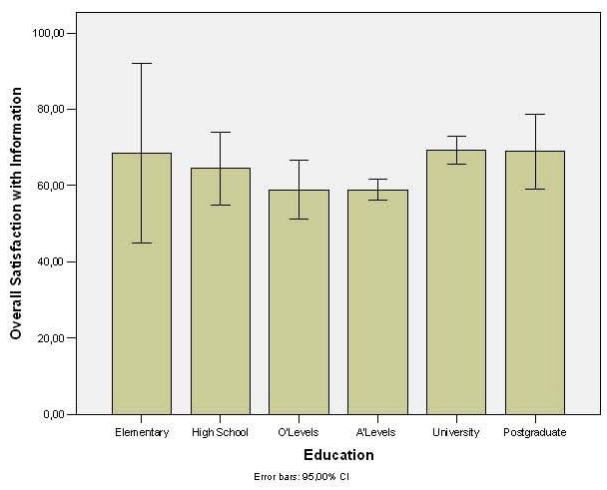
Association of education level with patient's overall satisfaction with the information received preoperatively.

Although gender (p = 0.7, t-test) and age (r = 0.04, p = 0.95) were not associated with the overall perceived quality of information, that was not the case for education levels: Patients with O level (a National exam in the UK sat at the age of 15 years of age) and A level education were least satisfied with the information they received, whilst those with Elementary or University education demonstrated the highest satisfaction rates (p = 0.01, One way ANOVA) (Figure 2)

### Extrinsic factors

Patients that were listed for surgery by a consultant had a trend toward higher overall satisfaction rates with the information received (mean 68.01, SD 15.7) and those by a Staff Grade or Specialist Registrar the lowest (mean 61.4, SD 18.5), although this difference was not statistically significant (p = 0.07, ANOVA). There was no significant difference in overall satisfaction with preoperative information between the patients undergoing major neck surgery and those undergoing minor surgery.

Overall satisfaction with information received was most closely correlated with the perceived quality of the information in the consent form (r = 0.58, p < 0.001), quality of the information sheets (r = 0.57, p < 0.001) and less so with self-obtained information (r = 0.49, p < 0.001). Information given by the GP (r = 0.38, p < 0.01), at the outpatient clinic (r = 0.39, p < 0.001) and at the preadmission clinic (r = 0.16, p = 0.02) were all significantly correlated with overall satisfaction with information received.

### Overall satisfaction with information received – multivariate analysis

It is entirely rational to suppose that information given at various stages was duplicated, and that the close correlation between the final outcome and that a particular source of information was a "spurious" association. In order to assess which sources of information were independently associated with the final outcome of a well informed patient, we performed stepwise multivariate linear regression: We included as dependent (outcome) variable the overall satisfaction with information obtained and as independent variables all other patient and doctor variables (including age, gender, educational level of the patient and grade of the doctor) as well as the perceived quality of the different information pathways.

A model of linear regression was built, describing the overall process of acquiring information: We found that only three factors predicted independently the final levels of patient information. These were: the perceived quality of information sheets (standardized coefficient 0.354, p < 0.001), self obtained information (standardized coefficient 0.349, p < 0.001) and information given by the ENT doctor at the outpatient appointment at the time of listing for surgery (standardized coefficient 0.151, p = 0.02). This three-factor model could explain more than 40% of the variability in the overall satisfaction with information received before the surgery (R square 0.42). It was notable that, although patients expressed relatively high levels of satisfaction with the information given at the preadmission clinic, the quality of the information they received there correlated poorly with how well informed overall they were about their operation.

## Discussion

In this study we found that patients are moderately satisfied with the overall information they receive prior to undergoing elective surgery (mean overall satisfaction rate: 6.3 in a scale of 0 to 10), but generally satisfied with the information they received in their outpatient appointment at the time of listing for surgery (mean 7.5) and at the preadmission clinic (mean 8.0).

Interestingly, although the information received at the pre admission clinic was rated as rather good, it was also found to be the least important predictor of overall satisfaction. Despite the poor correlation, the preadmission clinic is an important tool to reach a good level of information even with patients who are not overly pleased with the general information process. As expected however, the main onus of explaining the operation rests with the doctor who initially suggests to the patient to undergo surgery, i.e. the doctor seen at the outpatient clinic – and this was highlighted in our study.

Outcome studies have the benefit of assessing medical care from a patient perspective. However, they suffer from all the shortcomings of non blinded, non randomized studies: Although by maximizing response rates, we reduced the chance of non response bias, we could not avoid other forms of bias, especially in the case of questions referring directly to the service provided by the hospital team. However, this does not invalidate these questionnaires – it merely makes their interpretation more complex. In our case, we did not use this questionnaire to assess quantitively how satisfied were patients with our procedures, but mainly in order to understand the information process and all the factors in play.

Correlation analysis showed that patient perception of the quality of information received from different sources is closely linked. In other words, patients who report that they are satisfied with the information they received from the GP, also tend to find very useful the information they received at the outpatient clinic, the preadmission clinic and the information sheets etc. In order to exclude spurious associations, we used multivariate linear regression and built a model predicting overall satisfaction with information received. This showed that the quality of information sheets (standardized coefficient 0.354, p < 0.001), self obtained information (standardized coefficient 0.349, p < 0.001) and information given by the ENT doctor at the outpatient appointment (standardized coefficient 0.151, p = 0.02) at the time of listing for surgery were the most important factors at predicting overall satisfaction and could explain almost 40% of the overall satisfaction with information received. This confirms the complicated nature of the information process: many factors appear to play a part and some of these factors are outside the hospital. The doctor-patient consultation is only a small part (10–30 minutes) of this process. Waiting lists and the delay until the operation are additional factors that result in the patient frequently seeking information from other sources.

The importance of written information cannot be overstated: Patients receiving written information have significantly less anxiety preoperatively and less post operative pain [10] As the time spent with the patient decreases, and patients report decreased recall of the information received, they increasingly have to rely on written information that they can digest at their own time and which can serve as a reference. Similarly, increased use of self obtained information (other written sources as well as the internet) reflects an erosion of the paternalistic model of doctor – patient. The risk with this type of information is that it frequently is of variable quality, something which was also highlighted in our study: The patients graded it as the worst quality among all the information sources.

The doctors should not feel threatened by the the trend of patients to rely to information sources outside the hospital: On the contrary, they must acknowledge that guiding the patient to other sources (self help groups, internet sites, organizations) may be as important as time actually spent talking to the patient. Recognising this creates a common language with the patient and can help to bypass any feelings of antagonism.

It is of course of interest to know how we could tailor information-delivering pathways to specific patient groups. Our finding that the least satisfied patient group were those with relatively higher levels of education could be useful. The reason for this finding is not entirely clear, however, it means that we should engage these two patient groups to try and identify how we can improve their overall satisfaction with information provided. Patient discussion workshops could be used to try to improve the information given to these particular groups. Other sources of information such as digital compact discs and web based information could be considered.

## Conclusion

Overall patients are moderately satisfied (6.3/10) (overall quality of information, Table 1) with the information obtained/received before surgery. Although we should not underestimate the importance of the outpatient consultation, the importance of written material and non medical information sources was highlighted. It is up to us to understand and use these alternative information channels appropriately. We should also be looking ways to improve the provision of patient information, and where possible and appropriate, tailor it to specific patient groups.

## Abbreviations

GP: General Practitioner; ENT: Ear, Nose and Throat surgery; VAS: Visual Analoge Scale; SHO: Senior House Officer

## Competing interests

The authors declare that they have no competing interests.

## Authors' contributions

CG designed the study, did the statistical analysis and drafted the manuscript, EP participated in writing and editing the manuscript and GK participated in the organisation of the survey and acquisition of data.

## Pre-publication history

The pre-publication history for this paper can be accessed here:



## Supplementary Material

Additional file 1 Patient information questionnaire. The actual questionnaire submitted to the patients. Click here for file
