# Supplementary material for: The Information and Consent Process in Patients undergoing Elective ENT surgery: A cross-sectional survey
Source: BMC Ear Nose Throat Disord. 2008 Sep 17;8:5. doi: 10.1186/1472-6815-8-5 (PMC2556309; doi:10.1186/1472-6815-8-5)
Supplement: Additional file 1 — Patient information questionnaire. The actual questionnaire submitted to the patients. [file 1472-6815-8-5-S1.doc]

Appendix

Private and Confidential

Whipps Cross University Hospital / Department of Otolaryngology – Head and Neck surgery

**Patient Information how are we doing – how can we improve?**

Dear Sir/Madam,

You recently had an Ear, Nose and Throat operation. We are trying to find the best way of providing information about the procedures we perform. We would be grateful if you could guide us in this endeavor by completing this questionnaire. **All replies will be treated as strictly confidential.**

Your age _____ (years) Your Gender (please tick): Male ٱ Female ٱ

Your education (please tick one) Elementary ٱ High School ٱ O’Levels ٱ

A’Levels ٱ University ٱ Postgraduate Degree ٱ

Please grade the quality of information obtained, by marking with a pen on a scale of useless to excellent.

Information obtained by your **GP** (doctor or nurse practitioner)

Useless Excellent

By the **doctor you saw in your ENT appointment** (when you were told that you were going to have surgery)

Useless Excellent

By the **doctor you saw at the preadmission clinic** (when you came to specifically discuss your operation)

Useless Excellent

Information contained in the **information sheets** (brochure handed to you at the preadmission clinic)

Useless Excellent

Information in the **Consent form** (the form you signed to indicate that you agree to have surgery)

Useless Excellent

**Self obtained information (internet, journals, books, friends)**

Useless Excellent

**Finally, Overall you would say that the information you had before the operation was**

Useless Excellent

# Thank you for your contribution
